# Endometrial Cancer as a Metabolic Disease with Dysregulated PI3K Signaling: Shedding Light on Novel Therapeutic Strategies

**DOI:** 10.3390/ijms21176073

**Published:** 2020-08-23

**Authors:** Satoru Kyo, Kentaro Nakayama

**Affiliations:** Department of Obstetrics and Gynecology, Shimane University Faculty of Medicine, 89-1 Enya-cho, Izumo, Shimane 693-8501, Japan; kn88@med.shimane-u.ac.jp

**Keywords:** endometrial cancer, metabolic syndrome, tumor microenvironment, hyperinsulinemia, hyperglycemia, PI3K-AKT

## Abstract

Endometrial cancer (EC) is one of the most common malignancies of the female reproductive organs. The most characteristic feature of EC is the frequent association with metabolic disorders. However, the components of these disorders that are involved in carcinogenesis remain unclear. Accumulating epidemiological studies have clearly revealed that hyperinsulinemia, which accompanies these disorders, plays central roles in the development of EC via the insulin-phosphoinositide 3 kinase (PI3K) signaling pathway as a metabolic driver. Recent comprehensive genomic analyses showed that over 90% of ECs have genomic alterations in this pathway, resulting in enhanced insulin signaling and production of optimal tumor microenvironments (TMEs). Targeting PI3K signaling is therefore an attractive treatment strategy. Several clinical trials for recurrent or advanced ECs have been attempted using PI3K-serine/threonine kinase (AKT) inhibitors. However, these agents exhibited far lower efficacy than expected, possibly due to activation of alternative pathways that compensate for the PIK3-AKT pathway and allow tumor growth, or due to adaptive mechanisms including the insulin feedback pathway that limits the efficacy of agents. Overcoming these responses with careful management of insulin levels is key to successful treatment. Further interest in specific TMEs via the insulin PI3K-pathway in obese women will provide insight into not only novel therapeutic strategies but also preventive strategies against EC.

## 1. Introduction

Endometrial cancer (EC) is the most common gynecologic malignancy in the Western world. In the United States (US), EC accounts for 6.9% of cancer diagnoses in women, with an estimated 61,180 new cases and 12,160 deaths in 2019 [1]. In 10 years, the incidence of EC is expected to dramatically increase to over 120,000 cases, becoming the third most common malignancy affecting women in the US after lung and colorectal cancers [2]. In Japan, mortality from EC has dramatically increased by 2.3 and 4.3 times in the past two and three decades, respectively [3], presumably as a consequence of changes in the lifestyle in the Japanese population. Over 75% of women diagnosed with EC have stage I or II disease, and thus have good clinical outcomes with five-year overall survival of 75–90% [4,5]. Operative procedures and postoperative adjuvant treatments are well standardized, including hysterectomy with retroperitoneal lymphadenectomy, followed by paclitaxel/platinum-based conventional chemotherapies. However, women with recurrent or advanced disease have lower response rates to conventional treatments, and clinical outcomes are extremely poor [6,7,8,9]. Therefore, further therapeutic developments are needed for these patients, hopefully combined with molecular-targeted agents, based on novel aspects of precise molecular pathogenesis and carcinogenesis of this tumor.

EC is highly associated with a variety of metabolic disorders, including obesity, hypertension, and diabetes, known as the metabolic triad. The metabolic tumor microenvironments (TMEs) formed in these disorders are closely involved in the development of EC. In 2013, a new classification of EC was proposed by The Cancer Genome Atlas (TCGA) [10], based on the genomic status of the patients. TCGA analyses uncovered specific molecular landscapes including the dysregulated phosphoinositide 3 kinase (PI3K)-serine/threonine protein kinase B (AKT) pathway, which is essential for metabolic homeostasis and carcinogenesis of EC. This review article introduces the metabolic TMEs of EC, describes how the genomic status of EC and insulin-PI3K signaling direct TMEs, and suggests important clues for developing novel therapeutic and preventive strategies.

## 2. Reclassification of EC According to Genomic Features

### 2.1. Conventional Classification

Two pathogenetic types of ECs that are driven by distinct metabolic and endocrine signals were proposed in 1983 [11]. Type I tumors develop from a precursor lesion with atypical hyperplasia, and are the most commonly observed, usually representing low-grade endometrioid histology, with lower International Federation of Gynecology and Obstetrics (FIGO) stages [11]. This type is considered to be estrogen driven, to be associated with obesity, and to have a favorable clinical outcome. In contrast, type II tumors are relatively rare, represent high-grade serous histology, exhibit very aggressive tumor behaviors independent of estrogen, and are more common in nonobese women [11]. Type II tumors arise de novo, usually from atrophic endometrium in postmenopausal women, and have a considerably worse prognosis than type I tumors [11].

Histological assessment is subjectively assigned according to the microscopic appearance and predefined pathologic criteria. Tumor grade is defined by nuclear features and the proportion of glandular structures versus solid components, and the histologic subtype is determined by morphologic criteria and supported by immunohistochemistry. The pathologic accuracy of the tumor grade, especially in cases of grade 3 endometrioid and serous cancers, is occasionally low due to poor diagnostic reproducibility [12,13]. Interobserver disagreement on histologic subtype or tumor grade was reported in one-third or more of ECs [12,13]. Classical histologic classification is therefore insufficient to effectively triage patients for optimal treatment options. Objective information that mirrors the biological features as well as clinical behaviors of individual tumors is greatly needed.

### 2.2. Novel Molecular-Based Classification

Following these demands, TCGA reported the results of comprehensive genomic studies of ECs, including a combination of whole genome and exome sequencing, microsatellite instability (MSI), and copy number analyses [10]. Using this molecular information, a total of 232 endometrioid and serous ECs were classified into four groups (DNA polymerase epsilon (*POLE*) ultramutated, MSI hypermutated, copy-number low, and copy-number high) that correlate with progression-free survival.

#### 2.2.1. POLE Ultramutated Group

A novel category stated by TCGA is the ultramutated *POLE* subgroup, which consists of 7% of ECs with unusually high mutation rates (232 × 10^−6^ mutations/Mb), generating extremely frequent mutations in cancer-associated genes such as *PTEN* (94%), *PIK3CA* (71%), *PIK3R1* (65%), *FBXW7* (82%), *ARID1A* (75%), *KRAS* (53%), and *AIRD1B* (47%) [10]. This subgroup is characterized by mutations in the exonuclease domain of *POLE* and exemplified by an increased C →A transversion frequency [10]. *POLE* encodes the major catalytic and proofreading subunits of the Polε (polymerase epsilon) DNA polymerase complex, which incorporates bases with high fidelity during leading strand DNA replication, ensuring a low mutation rate in the daughter strand. Exonuclease domain mutations are mostly detected in hotspots and include P286R and V411L, which induce decreased proofreading abilities by this enzyme complex [10]. Eventually, increased rates of replicative errors occur, resulting in the ultramutated phenotype. To assess the *POLE* status, the original report used whole genome or exome sequencing, but subsequent studies used more focused methods including Sanger sequencing [14,15], target-gene panels [16,17], digital PCR [18,19], or functional analyses [20]. Very favorable clinical outcomes were shown in these studies in women with *POLE* mutations, even in those with high-grade tumors.

#### 2.2.2. MSI Hypermutated Group

TCGA described an MSI subgroup with high mutation rates (18 × 10^−6^ mutations/Mb). MSI represents a specific phenotype caused by a defective DNA mismatch repair (MMR) system composed of four representative MMR proteins (MLH1, MSH2, MSH6, and PMS2) [10]. MSI can be evaluated with PCR, which directly reflects the MSI status via amplifying a panel of five microsatellite marker DNA sequences based on Bethesda guidelines [21]. These markers have highly unstable mononucleotide repeat loci. When tumors have dysfunctional MMRs, PCR generates altered product sizes that reflect unstable microsatellites, compared to those from corresponding normal tissues [22]. More conveniently, MMR deficiency can also be detected by immunohistochemistry of MMR proteins [23]. MSI hypermutations can result from (a) an inherited cancer syndrome such as Lynch syndrome, which is characterized by germline mutations in MMR genes [24], (b) somatic mutations of these genes, or (c) epigenetic silencing such as DNA promoter methylation of these gene [23,25]. *MLH1* promoter hypermethylation is the leading cause of MSI in ECs [26]. MSI tumors account for 28% of ECs, and are characterized by approximately 10-fold greater mutation frequency than microsatellite-stable (MSS) tumors, few somatic copy number alterations (SCNAs), frequent *KRAS* mutations, high expression levels of phospho-AKT, and low expression levels of PTEN. PI3K pathway alterations are detected in 95% of the ECs in this group [10].

#### 2.2.3. Copy-Number High Group

This group contains primarily serous cancers with extensive SCNAs that were determined with Affymetrix SNP 6.0 microarrays using DNA from frozen tissue specimens and has a low mutation rate (2.3 × 10^−6^ mutations/Mb) [10]. Hierarchical clustering identified a group with significant reoccurring regions with amplifications or deletions, consisting of most serous cancers and one quarter of high-grade (grade 3) ECs [10]. The most striking feature of these tumors is the extremely high frequency of *TP53* mutations (detected in over 90% of patients) and a high frequency of *FBXW7* and *PPP2R1A* mutations [10], which were previously reported as common in serous cancers but not endometrioid cancer. Thus, a subset of high-grade endometrioid cancers have similar SCNAs and mutation spectra as serous cancers, indicating that they may benefit from treatment modalities used in patients with serous cancers. PI3K pathway alterations are detected in 60% of the ECs in this group, fewer than in the other three groups [10].

#### 2.2.4. Copy-Number Low Group

All remaining ECs that do not belong to the above three groups are categorized as the copy-number low group and mainly consist of MSS tumors. This group tends to have elevated progesterone receptor expression, suggesting potential responsiveness to hormone therapies. Copy-number low, MSS tumors have a high frequency of mutations in *CTNNB1* (52%), the only gene with a higher mutation frequency than in MSI tumors [10]. PI3K pathway alterations are observed in 92% of the ECs in this group [10].

Exome sequence data in TCGA study identified significantly mutated genes including *PTEN*, *PIK3R1*, *PIK3CA*, *FBXW7*, *KRAS*, and *POLE*, among which *PIK3CA* and *PIK3R1* mutations were most frequent [10]. However, unlike other tumor types, they co-occur with *PTEN* mutations in the MSI hypermutated and copy-number low groups. Of particular interest is that over 90% of endometrioid tumors have mutations in *PIK3CA*, *PIK3R1*, and *PTEN* [10], which encode the major components of the PI3K-AKT signaling pathway. Thus, a representative feature of EC is the much higher frequency of mutations in genes encoding components of the PI3K-AKT pathway than any other tumor type [10]. Most of these mutations cause enhanced signaling of this pathway and are tightly associated with metabolic TMEs, which are introduced in subsequent sections. The genomic landscape of cancer genomes has thus made the PI3K-AKT axis one of the most exploitable for drug development, especially for *POLE* mutated, MSI hypermutated, and copy-number low ECs. The *POLE* ultramutated and MSI hypermutated groups are associated with high neoantigen loads caused by an increased mutation rate along with increased infiltration of CD8-positive tumor-infiltrating lymphocytes [27,28,29]. Therefore, both types are considered to be good candidates for immune checkpoint inhibitor therapies. Taken together, novel molecular classification of ECs confers the theoretical background for optimal use of molecular-targeted therapeutics, according to the mutational status of individual ECs [29,30].

## 3. Risk Factors for EC

Among a variety of risk factors identified for EC, metabolic syndrome, which is a constellation of obesity, hypertension, hyperglycemia, and hyperlipidemia, has drawn great interest in the past two decades. The first case-control study for risk factors for endometrioid and serous types of EC was reported in 1997 [31]. Analysis of 328 endometrioid and 26 serous cancers demonstrated that body mass index (BMI), use of estrogen, age at menarche, and parity were significantly linked to the endometrioid subtype, but not serous cancers. The use of oral contraceptives was negatively associated with both subtypes. This study revealed the distinct risk factors associated with each histological subtype. Subsequent studies have accumulated consistent evidence demonstrating that metabolic syndrome is a representative risk factor for EC [21,22,23,24,25,26,27,28,29,30,31,32,33,34,35,36,37].

A meta-analysis of six studies [38] confirmed that metabolic syndrome is associated with an increased risk of EC (relative risk: 1.89, 95% confidence interval (CI) 1.34–2.67, *p* < 0.001), but with significant heterogeneity among the six studies (I^2^ = 92%, *p* < 0.001). The risk estimates for any single factor of metabolic syndrome were 2.21 (*p* < 0.001) for BMI, 1.81 (*p* = 0.044) for hyperglycemia, 1.81 (*p* = 0.024) for values of higher blood pressure, and 1.17 (*p* < 0.001) for high levels of triglyceride [38]. Another meta-analysis of 19 studies for EC [39] demonstrated that a BMI increase of 5 kg/m^2^ results in a 59% increased risk of EC (95% CI 1.50–16.8, *p* < 0.0001). A history of bariatric surgery and maintenance of normal weight after surgery were significantly associated with a 71 and 81% decreased risk for uterine cancers, respectively, including EC [40]. Although many studies have shown a positive association between a risk for EC and hypertension, hyperlipidemia, hyperglycemia, and diabetes mellitus, the risks from these factors were less robust than obesity [32,33,34,35,36,37,38,41,42]. A recent large-scale prospective study of 1205 women further confirmed that age (odds ratio (OR) 1.14, 95% CI 1.1–1.2) and BMI (OR 1.39, 95% CI 1.1–1.7) are positively associated with EC, but no significant association with other potential risk factors was found [43].

Recent progress in genome-wide association studies (GWAS) of EC have uncovered genetic risk regions [44], and the success of GWAS facilitated the use of Mendelian randomization methods to examine the risk factors of EC. Mendelian randomization utilizes genetic variants associated with a candidate risk factor as “instruments” in an instrumental variable analysis and examines the association of the risk factor without confounding factors [45]. The initial Mendelian randomization analysis used the single nucleotide polymorphisms associated with BMI (97 variants) as instrumental variables [46], and EC risk was found to be higher in individuals with genetically predicted higher BMI (OR 1.13, 95% CI 1.04–1.22, *p* = 0.002). A subsequent analysis also found higher EC risk with genetically predicted higher BMI (OR 2.11, 95% CI 1.94–2.28, *p* = 3.4 × 10^−17^) [47]. These studies clearly confirmed the causal relationship between obesity and EC.

## 4. Molecular Mechanisms Linking Obesity and EC

### 4.1. Excess Estrogen Production in Adipose Tissues

The endometrium is a highly regenerative tissue, and its proliferative activity is strictly controlled by estrogen and progesterone in each menstrual cycle. Long-term estrogen exposure without progesterone promotes the development of EC [48]. In postmenopausal women, ovarian function to produce these hormones is greatly decreased, and systemic and local hormonal levels significantly decrease. However, EC is primarily a disease of postmenopausal woman, with about 25% of cases occurring in premenopausal women and only 5% occurring in women younger than 40 years of age [49].

Why does obesity increase the risk of EC? In postmenopausal women, estrogen is no longer supplied by the ovaries, but by adipose tissues via the conversion of androstenedione to estradiol by aromatase. Aromatase activity is higher in obese women than nonobese women, presumably due to higher amounts of adipose tissues capable of expressing aromatase [50,51]. The concentration of estrogen is higher in adipose tissues than serum [52], and adipose tissues are the main source of estrogen. Adipose tissue is also the most common metastatic site of EC. Thus, the higher estrogen levels in obese women increase the risk of EC. A Mendelian randomization analysis using the genetic variants highly associated with serum estrogen levels in postmenopausal women confirmed the causal relationship between serum estrogen levels and EC (OR 1.15, 95% CI 1.11–1.21, *p* = 4.8 × 10^−11^) [53]. In relation to estrogen levels, older age at menarche (that would be presumed to decrease lifetime estrogen exposure) was confirmed to be associated with a lower risk of EC (OR 0.78, 95% CI 0.70–0.87, *p* = 1.0 × 10^−5^) [54].

**Figure 1:**
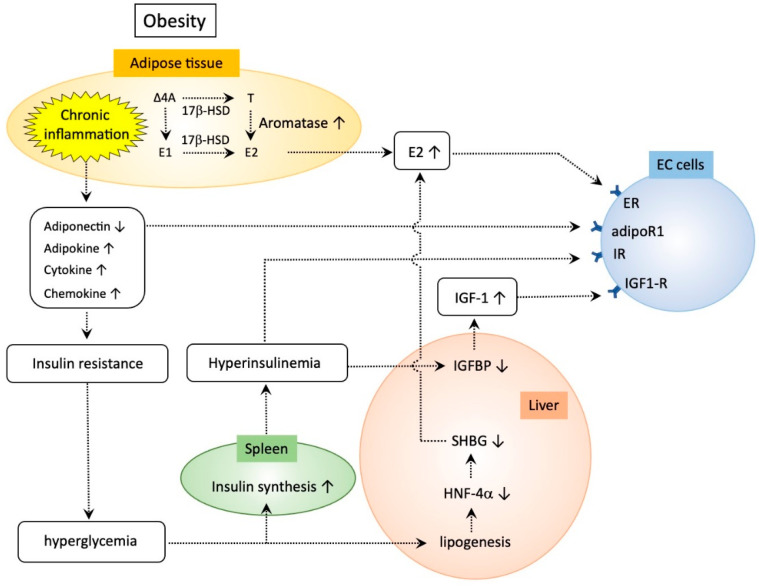
Potential mechanisms of obesity-induced development of endometrial cancer (EC). Obesity induces increased aromatase expression and chronic low-grade inflammation in adipose tissues that play fundamental roles in EC development. The former results in increased estradiol (E2) production, and the latter triggers expression of various adipokines, cytokines, and chemokines, leading to insulin resistance. Insulin resistance causes hyperglycemia, which induces insulin synthesis in the spleen and lipogenesis in the liver. The former subsequently induces hyperinsulinemia, and the latter causes decreased expression of sex hormone-binding globulin (SHBG), leading to enhanced E2 activity. Hyperinsulinemia plays central roles in promoting EC growth via decreased insulin-like growth factor binding protein (IGFBP) 1 with enhanced insulin-like growth factor (IGF)-1 activity and most importantly, switching on of insulin-phosphoinositide 3 kinase (insulin-PI3K) signaling in EC cells. Δ4A: androstenedione, 17β-HSD: 17β-Hydroxysteroid dehydrogenases, T: testosterone, ER: estrogen receptor, adinoR1: adiponectin receptor 1, IR: insulin receptor, IGF1-R: insulin-like growth factor 1-receptor, HNF-4α: hepatocyte nuclear factor-4α. ↑ (up regulation), ↓ (down regulation), ⇢ (expected pathway).

Sex-hormone-binding globulin (SHBG) is a circulating sex steroid transporter secreted by the liver that binds to circulating sex steroids with high affinity and negatively regulates the concentration of bioactive sex hormones in the blood, affecting the bioavailability of sex steroids including estrogen [55]. Low serum levels of SHBG are associated with metabolic syndrome [56]. Diets leading to weight loss in women are associated with an increase in SHBG levels [57]. Exposure of hepatic cells to glucose significantly reduces *SHBG* mRNA expression via decreased hepatocyte nuclear factor-4 alpha (HNF-4α), which is the key transcription factor that binds the *SHBG* promoter and is down-regulated by glucose-induced de novo lipogenesis in the liver [58]. Eventually, down-regulation of SHBG in obese women leads to enhanced bioavailability of estrogen. Taken together, these findings indicate that obesity increases the risk of EC via increasing systemic and local estrogen activity, a notable characteristic of TMEs in EC, strongly accounting for the increased risk in obese women (Figure 1).

### 4.2. Chronic Low-Grade Inflammation in Adipose Tissues

Although originally classified as a simple energy storage organ, adipose tissues comprise a variety of cell types including adipocytes, immune cells, endothelial cells, and fibroblasts [59]. However, they are currently considered to function as a major endocrine system that produces and releases diverse types of secretory proteins, adipokines, growth factors, cytokines, and chemokines into the systemic circulation [60]. In obese women, excessive accumulation of adipose tissues accompanies inflammatory changes, leading to chronic low-grade inflammation characterized by increased macrophage infiltration and mildly elevated circulating adipokines (leptin, adiponectin, resistin, visfatin, omentin), cytokines (interleukin (IL)-6, tumor necrosis factor (TNF)-α), chemokines (macrophage chemoattractant protein (MCP)-1), acute phase reactants (C reactive protein), plasminogen activator inhibitor-1, and serum amyloid A [61,62,63,64] (Figure 1). This type of inflammation in obesity is fundamentally distinct from typical inflammatory responses that are induced as host defense, because obesity-related inflammation is sterile, mild, low-grade, and most importantly, causes insulin resistance. Adipokines are categorized as pro- or anti-inflammatory adipokines according to their effects on inflammatory responses in adipose tissues. Most of them are proinflammatory, whereas adiponectin is anti-inflammatory. Low serum levels of adiponectin are associated with obesity, insulin resistance, and hyperinsulinemia [65]. In patients with EC, serum levels of adiponectin are significantly decreased compared with those with benign disorders or normal endometrium [66]. Serum levels of adiponectin are negatively correlated with the risk of EC, especially in postmenopausal women [67]. EC cells express adiponectin receptors, and adiponectin can directly suppress proliferation of EC via adiponectin-mediated AMP-activated protein kinase (AMPK) activation [68]. A recent meta-analysis of 18 studies examined the association of various circulating adiponectin and cytokine levels and revealed that patients with circulating adiponectin levels in the highest tertile had a decreased EC risk compared to those with levels in the lowest tertile (OR 0.51, 95% CI 0.38–0.69, *p* < 0.00001). Women with circulating leptin concentrations in the highest tertile showed an increased EC risk compared to those with concentrations in the lowest tertile (OR 2.19, 95% CI 1.45–3.30, *p* = 0.0002) [69].

### 4.3. How Does Chronic Inflammation Induce Insulin Resistance?

The molecular mechanisms through which the obesity-induced inflammatory response cause insulin resistance have recently been investigated [70,71]. Insulin resistance is defined as the perturbation of insulin-mediated signaling pathways, leading to systemic hyperglycemia. The components of these pathways are directly or indirectly targeted by obesity-induced inflammatory responses.

Zhan et al. studied the effect of TNF-α on insulin resistance in adipocytes and hepatocytes [72]. Treatment with TNF-α leads to phosphorylation of S6 kinase 1 (S6K1), a serine/threonine kinase downstream from AKT in the insulin signaling pathway that is involved in negative feedback regulation of insulin action. This TNF-α-induced phosphorylation of S6K1 triggers the phosphorylation of insulin receptor substrate (IRS)-1 at multiple serine residues, resulting in accelerated degradation of IRS-1 and impaired insulin-stimulated glucose uptake in adipocytes.

Kamei et al. demonstrated the effects of MCP-1 on insulin resistance using MCP-1 transgenic mice [73]. Overexpression of MCP-1 in adipose tissue results in systemic insulin resistance and decreased tyrosine phosphorylation of the insulin receptor (IR) and IRS-1. This phenomenon was concurrent with decreased phosphorylation of AKT, which was partially restored by mitogen-activated protein kinase inhibitors, indicating that the extracellular signal-regulated kinase pathway is part of the mechanism by which MCP-1 inhibits insulin signaling.

Hirosumi et al. demonstrated that c-Jun amino-terminal kinase (JNK) activity is abnormally elevated in obesity [74]. TNF-α-induced phosphorylation of IRS-1 at Ser 307 leads to insulin resistance in liver cells, and a JNK inhibitor largely abolishes the phosphorylation of IRS-1 at Ser 307 and restores insulin resistance, indicating that JNK activity is involved in the mechanism of TNF-α-induced insulin resistance.

Rotter et al. examined the effect of IL-6 on insulin resistance [75]. Treatment of adipocyte cells with IL-6 does not increase phosphorylation of IRS-1 at Ser 307, but exerts long-term inhibitory effects on the gene expression of IRS-1. Similarly, IL-6 inhibits gene expression of glucose transporter (GLUT)-4 as well as peroxisome proliferator-activated receptor gamma, a factor that increases insulin sensitivity by enhancing storage of fatty acids in fat cells and upregulates adiponectin release from these cells. Importantly, the expression of IL-6, like that of TNF-α and IL-8, is markedly increased in adipocytes from insulin-resistant individuals. Jager et al. examined the effect of IL-1β on glucose uptake of adipocytes [76] and found that prolonged IL-1β treatment reduces insulin-induced glucose uptake with marked inhibition of GLUT-4 translocation to the plasma membrane in response to insulin. This inhibitory effect involves a decrease in the amount of IRS-1 mRNA and protein. Taken together, multiple cytokines coordinately participate in the development of insulin resistance in adipose tissues (Figure 1).

### 4.4. Role of the Insulin-Like Growth Factor (IGF)–IGF Binding Protein (IGFBP) Axis in the Development of EC

IGFs are growth factors that modulate steroid hormone activity in the endometrium via autocrine and paracrine regulatory loops. Endometrial stromal cells are the major source of IGF-I and IGF-II, and epithelial cells mainly produce IGF receptors [77]. The IGF axis is essential for not only endometrial physiology but the pathogenesis of EC. Obesity-induced insulin resistance presumably leads to hyperglycemia, leading to increased synthesis of insulin. Eventually, upregulated insulin levels lead to inhibition of the synthesis of IGFBP-1 [78,79,80]. IGFBP-1 binds to IGF to stabilize the complex, and excess amounts of IGFBP-1 reduce the bioavailability of IGF to their receptors and suppress subsequent intracellular IGF signaling [80]. Thus, insulin-induced suppression of IGFBP-1 results in enhanced biological activity of IGF. Ayabe et al. found an increase in circulating levels of IGF-1 and a decrease in IGFBP-1 in postmenopausal women with EC [81]. Studies by several groups have shown that IGF-1 plays a role in the development of both Type I and II ECs, emphasizing the importance of altered *IGF1R* expression [82,83,84]. Estrogen enhances IGF-1 activity in EC cells [85,86], and increased local estrogen levels in obese women may therefore stimulate IGF-1 synthesis in the endometrium [80]. Collectively, obesity-induced insulin resistance enhances IGF-1 activity in concert with the excess estrogen locally produced in obese women, leading to development of EC (Figure 1).

## 5. Is Diabetes Mellitus No Longer a Risk Factor for EC?

Several meta-analyses support the idea that diabetes is associated with an approximately two- to threefold increased risk of EC [41,87,88] independent of obesity [41,89,90,91]. A large-scale case-control study indicated a twofold increase in the risk of EC in women with diabetes [34]. However, some cohort studies showed that such an association is lost or weakened to modest levels when adjustments are made for BMI [92,93]. Therefore, the relationship between diabetes and EC is still controversial.

Seven representative large-scale cohort studies measured the blood glucose levels and evaluated the risk of EC [32,33,94,95,96,97,98]. Most studies demonstrated that women with higher baseline glucose levels have a 1.2–2.6 times increased risk for EC. However, the strength of such an association varies depending on age, BMI, and menopausal status, and one cohort study demonstrated that blood glucose levels are not associated with EC [93]. Five large-scale case-control studies demonstrated significantly higher blood glucose levels in women with ECs than controls [37,99,100,101,102].

In a Mendelian randomization analysis [103], single nucleotide polymorphisms associated with diabetes (49 variants), fasting glucose (36 variants), fasting insulin (18 variants), and early insulin secretion (17 variants) were used to examine the association with the risk of EC. EC risk was higher in individuals with genetically predicted higher fasting insulin levels (OR 2.34, 95% CI 1.06–5.14, *p* = 0.03) or genetically predicted higher 30 min postchallenge insulin levels (OR 1.40, 95% CI 1.12–1.76, *p* = 0.003). Importantly, however, no associations were observed between genetic risk for diabetes (OR 0.91, 95% CI 0.79–1.04, *p* = 0.16) or higher fasting glucose (OR 1.00, 95% CI 0.67–1.50, *p* = 0.99) and EC. This study thus supports a causal association between higher fasting insulin levels and a risk for EC, independent of higher glucose levels. Collectively, these studies indicate that hyperinsulinemia, rather that hyperglycemia, is more directly associated with a risk for the development of EC.

## 6. Roles of Insulin-PI3K Signaling Pathways in Metabolic TMEs

### 6.1. The Insulin-PI3K Signaling Pathway

**Figure 2:**
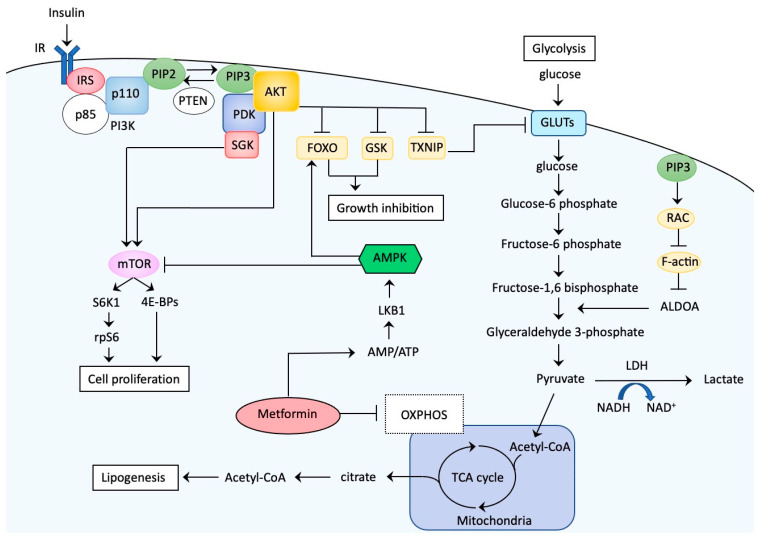
Insulin-PI3K signaling facilitates cell proliferation and glucose metabolism via serine/threonine protein kinase B (AKT)-dependent and -independent mechanisms. The interaction of insulin with the insulin receptor (IR) triggers phosphoinositide 3-kinase (PI3K)-regulated signaling, in which phosphatidylinositol 3,4,5-triphosphate (PIP_3_) recruits phosphoinositide-dependent kinase 1 (PDK1), the serine/threonine kinase AKT, and other factors to the cell membrane where the signals are propagated via a series of serine/threonine kinase and tyrosine kinase activities. Phosphatase and tensin homologue (PTEN) can dephosphorylate PIP3 to form PIP2, limiting activation of this pathway, and loss-of-function mutations in *PTEN* cause significant elevations in PIP3, a driving force of this pathway. Phosphorylated AKT inhibits downstream targets via phosphorylation, including forkhead box protein O (FOXO)-1 and glycogen synthase kinase (GSK), both of which normally function to inhibit cell growth. Phosphorylated AKT also inhibits thioredoxin-interacting protein (TXNIP), an adaptor for endocytosis of glucose transporters (GLUTs), leading to dissociation from the GLUTs, inhibition of their endocytosis, and rapid glucose uptake upon insulin stimulation. AKT phosphorylation leads to activation of mammalian target of rapamycin (mTOR), a protein kinase that controls cell proliferation via activation of the eukaryotic initiation factor 4E-binding protein-1 (4E-BP-1) complex and S6 kinase 1 (S6K1), which phosphorylates ribosomal S6 protein (rpS6). Insulin-PI3K signaling also activates an AKT-independent pathway, in which PIP_3_-recruited Ras-related C3 botulinus toxin substrate (RAC) activation leads to disruption of the actin cytoskeleton and release of filamentous actin (F-actin)-bound aldolase A (ALDOA), resulting in aldolase activation that promotes the glycolysis pathway. Additionally, PI3K signaling activates serum and glucocorticoid-induced protein kinase (SGK1) via PDK phosphorylation independent of AKT and can phosphorylate overlapping substrates of AKT, resulting in bypass of the AKT pathway. Metformin inhibits oxidative phosphorylation (OXPHOS) at the mitochondrial level, resulting in a decrease in the proton gradient across the inner mitochondrial membrane and leading to a reduction in proton-driven synthesis of adenosine triphosphate (ATP) and an increase in the ratio of cellular adenosine monophosphate (AMP) to ATP. This leads to preferential AMP binding to AMP-activated protein kinase (AMPK) and a conformational change that allows for phosphorylation/activation of AMPK by liver kinase B1 (LKB1). Activated AMPK converts cells to a catabolic state through AMPK-mediated phosphorylation, leading to the inhibition of downstream transcription factors involved in ATP-consuming synthetic pathways. AMPK activation also induces FOXO1 nuclear translocation, activating function of this protein. Additionally, activated AMPK inhibits mTOR to inhibit cell growth. IRS: insulin receptor substrate, p85: regulatory subunit of PI3K, p110: catalytic subunit of PI3K, NAD: nicotinamide adenine dinucleotide, LDH: lactate dehydrogenase, Acetyl-CoA: acetyl coenzyme A, TCA: tricarboxylic acid. → (activation), ⊣ (inhibition).

The insulin-PIK3 signaling axis plays a fundamental role in human cellular growth as well as glucose metabolism [104,105] (Figure 2).

The details of the signaling pathway are shown in the representative review articles [106,107]. At the cell surface, insulin binds to the IR, which exists as an α_2_β_2_ heterodimer. Following insulin binding, the tyrosine kinase domain of the β subunit autophosphorylates the subunit and has intrinsic kinase activity on proximal substrates such as IRS family proteins. Phosphorylation of IRS1/2 on specific tyrosine residues can lead to interactions with the regulatory subunit of PI3K (p85) and then inhibit the catalytic subunit (p110) of PI3K, allowing it to phosphorylate phosphatidylinositol 4,5-bisphosphate (PIP_2_). The product of this reaction is a second messenger, phosphatidylinositol 3,4,5-triphosphate (PIP_3_). Inversely, phosphatase and tensin homologue (PTEN) can dephosphorylate PIP3 to form PIP2, limiting activation of this pathway. Loss-of-function mutations of *PTEN* cause significant elevations in PIP3, a driving force of this pathway. The newly produced PIP_3_ recruits the following factors to the cell membrane: phosphoinositide-dependent kinase 1 (PDK1), AKT, and other factors with a pleckstrin homology domain, a shared property of these PI3K effectors. This process involves propagation of signals via a series of serine/threonine kinases, tyrosine kinases, and modulators with small GTPase activities, resulting in activation of multiple diverging downstream pathways that confer a large portion of the metabolic functions of insulin. AKT is more universally downstream of receptor-mediated PI3K activation. Through colocalization of the constitutively active PDK with AKT at the plasma membrane via the pleckstrin homology domain, PDK1 mediates phosphorylation of AKT. Phosphorylated AKT further phosphorylates several downstream targets, including thioredoxin-interacting protein (TXNIP), glycogen synthase kinase (GSK), and forkhead box protein O1 (FOXO1) [108,109]. TXNIP phosphorylation results in inhibition of its function. Because TXNIP functions as an adaptor for endocytosis of GLUTs, TXNIP inactivation via phosphorylation leads to dissociation from the GLUTs, thus inhibiting their endocytosis and resulting in rapid glucose uptake upon insulin stimulation. AKT also phosphorylates GSK3, which results in inhibition of GSK3 activity and activation of glycogen synthesis in muscle and liver. AKT phosphorylation of FOXO1 causes translocation of FOXO1 from the nucleus to the cytoplasm, leading to the failure of FOXO1 to transactivate downstream targets that negatively regulate cell proliferation. AKT phosphorylation leads to activation of mammalian target of rapamycin (mTOR), a protein kinase that controls cell proliferation via activation of the eukaryotic initiation factor 4E-binding protein-1 complex and S6K1, which phosphorylates the ribosomal S6 protein. Although AKT is a universal downstream target of receptor-mediated PI3K activation, alternative AKT-independent signaling cascades are initiated by PI3K activity and impact glucose metabolism. Representative ATK-independent pathways involving glycolysis and cell proliferation are conferred by Ras-related C3 botulinus toxin substrate (RAC) or serum and glucocorticoid-induced protein kinase (SGK) signaling [104,105,110].

### 6.2. Status of the PI3K-AKT Pathway Affects Tumor Behavior via Altered Glucose Metabolism

Tumors have modified cellular metabolism based on aerobic fermentation, with a propensity to shift away from the oxidative phosphorylation pathway to the anabolic glycolysis pathway in which the final product of glycolysis, pyruvate, is converted into lactate [111,112]. In the anabolic glycolysis pathway, tumor cells generate far fewer adenosine triphosphate (ATP) molecules per molecule of glucose than normal cells that use the oxidative phosphorylation pathway. Therefore, tumor cells require greater glucose uptake than normal cells [113]. This increase in glucose flux is largely mediated by insulin-PI3K signaling. Mutations, mostly gain-of-function mutations, in the genes that encode components of this pathway result in enhanced signaling activity. Therefore, insulin plays critical roles in tumor growth via the PI3K signaling pathway. This concept is supported by studies demonstrating insulin-mediated acceleration of tumor development in humans and mouse models [114,115]. In patients with EC, serum levels of insulin are elevated with concomitant up-regulation of IR, IRS-1, and AKT expression compared to those without EC [116]. Levels of p-IR, p-IRS-1, and p-AKT are higher in EC patients with advanced stage disease, high-grade tumors, myometrial invasion, and lymph-node metastasis, and are significantly correlated with levels of serum insulin. In EC cells, insulin-induced mitogenic effects are inhibited by treatment with LY294002, a PI3K inhibitor [116]. Thus, insulin plays an essential role in the tumorigenesis of EC via the PI3K-AKT pathway, consistent with accumulating epidemiological studies indicating that hyperinsulinemia, rather than hyperglycemia, plays essential roles in the development of EC, as discussed above.

The tumorigenic effects of insulin depend on the genotype in tumors. A study with human cancer cell lines in a mouse xenograft model demonstrated that tumors resistant to dietary restriction carried mutations that constitutively activate the PI3K signaling pathway [117]. Substitution of an activated mutant allele of *PI3K* with wild-type *PI3K* in otherwise isogenic human cancer cells successfully converted a tumor resistant to dietary restriction to one that was sensitive to dietary restriction [117]. Restoration of PTEN expression in a PTEN-null cancer cell line also showed similar conversion. In engineered mouse tumors, dietary restriction did not affect the tumor burden of PTEN-null mouse tumors, but it significantly decreased the size of tumors that lacked constitutively activated PI3K signaling [116]. Thus, the PI3K pathway is key to the sensitivity of tumors to dietary restriction, and different levels of PI3K activation in tumors contribute to their differential sensitivities to dietary restriction [118].

The frequency of genetic alterations in PIK3 signaling pathways varies among tumor types, and EC is the leading type of cancer, with over 90% of cases harboring alterations in the pathway [10,119,120]. In tissues with chronic exposure to high levels of insulin such as the pancreas and liver, PI3K signaling is constitutively active, and genetic alterations may not be required to maintain metabolic environments [107]. In contrast, the endometrium probably has low or normal levels of insulin signaling that may be insufficient to maintain the high regenerative capacity [107]. Increasing insulin sensitivity by acquired mutations of PI3K pathway genes may overcome this strict environment, conferring an insulin-dependent growth advantage with optimal TMEs. This may be one reason why EC has extremely frequent genetic alterations in PI3K pathways. In the circumstances of obesity-induced insulin resistance and hyperinsulinemia, genetic alterations in PI3K pathways that enhance signal intensity may cause further exaggeration of signaling, promoting tumor development. In this regard, PI3K signaling seems to be an attractive target for molecular-based therapies against EC.

## 7. What about Metformin? Is the PI3K-AKT Pathway a Target?

Metformin is a biguanide that is commonly used to treat diabetes. Recent studies have accumulated evidence showing potential benefits of metformin on the incidence, clinical outcome, and survival of EC, although contradictory results have been reported, especially for survival [121]. The direct antitumor effects of metformin are mainly due to via activation of the AMP-activated protein kinase (AMPK) pathway as well as inhibition of the PI3K-AKT pathway (Figure 2). Initially, metformin inhibits oxidative phosphorylation at the mitochondrial level, causing a reduction in the proton gradient across the inner mitochondrial membrane, with a decrease in proton-driven synthesis of ATP and an increase in the ratio of cellular adenosine monophosphate (AMP) to ATP. Eventually, AMP preferentially binds to AMPK with a subsequent conformational change that allows for phosphorylation/activation of AMPK by liver kinase B1 (LKB1) [117]. Activated AMPK converts cells to a catabolic state through AMPK-mediated phosphorylation, leading to inhibition of downstream transcription factors involved in ATP-consuming synthetic pathways. Additionally, activated AMPK inhibits mTOR, leading to decreased phosphorylation of the eukaryotic initiation factor 4E binding protein-1 complex, S6K1, and ribosomal S6 protein, all of which cause reduced protein synthesis [122]. Thus, metformin can also be classified as an mTOR inhibitor.

Furthermore, metformin decreases secretion of IGF-1 and expression and phosphorylation of IGF-1R, whereas it increases expression of IGFBP-1 in EC cells [123,124,125,126,127,128,129,130,131,132,133,134,135]. Thus, metformin targets the PI3K-AKT pathway.

A recent study also showed that FOXO1 is another target of AMPK. AMPK activation induces FOXO1 nuclear translocation and activates function of this protein. An in vitro study using EC cells reported that metformin-induced nuclear accumulation of FOXO1 is concurrent with an AMPK-dependent decrease in phosphorylated FOXO1 and transactivates the downstream target, leading to inhibition of cell proliferation [126].

Thus, although metformin has been traditionally and widely used to treat diabetes, it may be effective in EC by targeting both the AMPK and PI3K-AKT pathways. Several retrospective observational studies of metformin were reported for EC [127,128,129,130,131,132]. These studies compared the survival of metformin users with that of nonusers in diabetic or nondiabetic, type I or type II EC patients, and the results were contradictory; some studies showed greater survival in metformin users than nonusers, whereas others failed to find any effect on survival parameters, possibly due to high heterogeneity in patients. Nevertheless, a meta-analysis of the above studies supports a greater overall survival in metformin users compared to nonusers [133], although the low number of studies and the lack of randomized studies compromise the evidence level of the meta-analysis. Clinical trials using metformin for atypical endometrial hyperplasia (AEH) or EC have recently accumulated evidence for its efficacy [134,135,136,137,138,139,140,141,142,143,144,145,146]. Most studies used the presurgical window approach, in which patients diagnosed by endometrial biopsy were treated with metformin during the period prior to hysterectomy, and some studies targeted patients who underwent fertility-sparing treatments in combination with progestins. The results are summarized in Table 1.

Most studies evaluated tumor and serum factors in relation to proliferative activity or insulin signaling, and some factors were affected by metformin as expected. Notably, one recent randomized controlled trial of fertility-sparing treatment evaluated the clinical benefit in AEH or EC patients, and found that the complete response rate within 16 weeks of treatment was higher in the metformin plus megestrol group than in the megestrol group (34.3 versus 20.7%, OR 2.0, 95% CI 0.89–4.51, *p* = 0.09), and the difference was more significant in AEH patients (39.6 versus 20.4%, OR 2.56, 95% CI 1.06–6.21, *p* = 0.04) [146]. Few clinical trials have been reported for advanced or recurrent ECs, but a phase 2/3 trial by the Gynecologic Oncology Group for advanced or recurrent EC (NCT02065687) is ongoing and is comparing paclitaxel/carboplatin alone or in combination with metformin as first-line chemotherapy. Furthermore, a phase 2 trial of metformin for advanced and recurrent EC is ongoing and is testing the combination of letrozole and everolimus (NCT01797523), and a phase 1/2 trial of metformin combined with metronomic cyclophosphamide and olaparib (NCT02755844) is also underway.

## 8. Pitfalls of Targeting PI3K-AKT Pathways for the Treatment of EC

### 8.1. Clinical Outcome of Treatment of EC with PI3K-AKT Inhibitors

Accumulating preclinical studies have clearly demonstrated that insulin signaling greatly affects the growth properties of EC in different metabolic conditions. Targeting insulin signaling has a theoretical background in EC because EC has an extremely high frequency of genetic abnormalities in genes that encode components of the PI3K-AKT pathway, leading to enhanced insulin signaling. Several clinical trials have been performed for recurrent or advanced EC with molecular-targeting agents that target components of PI3K-AKT pathways [147,148,149,150,151,152,153,154,155,156,157] (Table 2). Response rates of these studies were disappointing, at less than 20%, although all studies enrolled patients with refractory ECs who were mostly pretreated with multiple chemotherapy regimens. Notably, some of these studies enrolled patients with mutations in genes that encode components of the PI3K-AKT pathway to examine whether these patients would benefit from these agents. However, most studies failed to find superior responses in such patients compared to those with wild-type genotypes.

### 8.2. Potential Molecular Mechanisms of Treatment Failure

Considering the frequent genetic alterations in components of the PI3K-AKT pathway, we expected superior effects of these inhibitors in EC. How can we explain these unexpected results? One possible explanation is that EC is not a type of tumor that exhibits oncogene addiction such as *ALK*-translocated non-small-cell lung cancers, and EC with *PIK3CA* mutations may not always exclusively depend on PI3K-AKT signaling for growth [105,158]. Even in tumors that are highly dependent on the oncogenic program conferred by mutations in components of the PI3K-AKT pathway, alternative pathways can be activated for tumor survival, overcoming the acute inhibitory actions of PI3K-AKT inhibitors [159]. Furthermore, PI3K signaling may activate the AKT-independent pathway in some ECs [105], and AKT inhibitors are not expected to exert effects in such tumors. For example, SGK family members are closely related to AKT with common upstream signaling and downstream targets, and EC has significantly increased SGK1 activity compared to normal endometria [160]. In tumors in which AKT is not constitutively activated or inhibited by drugs, PI3K signaling activates SGK1 and may phosphorylate overlapping substrates, resulting in bypass of the AKT pathway [158] (Figure 2). Levels of SGK1 expression can be used as markers to predict the efficacy of AKT inhibitors [160]. Thus, further refining patient populations with specific molecular markers that predict efficacy is of particular importance, and exploitation of reliable markers should be a strong focus. Finally, a limitation of the use of PI3K-AKT inhibitors is that multiple adaptive mechanisms may limit the efficacy of these agents in which insulin feedback mechanisms play critical roles [161]. In healthy metabolic tissues including liver, skeletal muscle, and adipose tissues, the insulin-PI3K pathway coordinates the clearance of glucose from the blood. Once this pathway is disrupted, the tissues perceive changes in signaling as a decrease in insulin levels. When this happens, the liver responds by increasing the release of glucose, leading to elevated levels of blood glucose and concomitant release of insulin from the pancreas. Eventually, induced hyperinsulinemia exerts tumorigenic effects. In fact, reflex hyperglycemia and hyperinsulinemia were observed when PIK3 was targeted with PI3K inhibitors [162,163] (Table 2). Patients with diabetes are usually excluded from clinical trials using PI3K-AKT inhibitors, but patients with potential insulin resistance, including those who are borderline diabetic, may be enrolled. Patients exhibiting hyperglycemia during treatment with PI3K-AKT inhibitors probably used exogenous insulin to reduce blood levels of glucose, possibly leading to therapy-induced hyperinsulinemia. The insulin feedback system may therefore be a limitation when inhibitors targeting insulin signaling are used.

### 8.3. How Can Drug Resistance Be Overcome to Improve the Efficacy of PI3K-AKT Inhibitors?

Concomitant inhibition of IR with PI3K-AKT inhibitors may be a potential strategy for preventing the unfavorable effects of reflex hyperglycemia and hyperinsulinemia. However, this is not a simple strategy. Some studies selected IGF-1R inhibitors as a therapeutic strategy for treating a variety of tumors in combination with IR inhibitors [164]. Although such combinatorial treatments showed some enhanced antitumor activity, extreme hyperglycemia and hyperinsulinemia occurred, which can reactivate IR and affect long-term treatment outcomes [165,166]. Thus, targeting insulin signaling requires further modalities.

A variety of approaches have been proposed for minimizing insulin levels. As a pharmacological approach, inhibitors of sodium-glucose co-transporter 2 (SGLT2) are potential candidates that can reduce serum levels of glucose by inhibiting reabsorption of glucose from the renal ultrafiltrate, resulting in clearance of glucose from the serum into urine. The combination of SGLT2 inhibitors with PI3K inhibitors successfully prevents reflex hyperglycemia and hyperinsulinemia [160,167]. Potassium channel inhibitors such as diazoxide may also be an alternative pharmacological approach to combine with PI3K inhibitors to inhibit release of insulin from the pancreas [168,169].

More than one third of the population in the USA are thought to have some degree of insulin resistance. In such individuals, consideration of the pivotal role of diet and exercise in reducing the glycogen stock and preventing therapy-induced hyperglycemia and hyperinsulinemia may be important [107,162,170]. At present, no consistent evidence in humans exists to suggest that a combination of diet or exercise with PI3K-AKT inhibitors reduces serum levels of insulin and improves the efficacy of treatment for EC. Clinical trials will therefore be needed to test these strategies in a clinical setting. Currently, 11 combination trials are ongoing in which the ketogenic diet is combined with standard chemotherapy or radiotherapy to examine the feasibility of these diets to treat patients with cancer [107]. Because EC is a leading cancer that is tightly associated with metabolic disorders and a dysregulated PI3-AKT pathway, the fundamental role of diet, exercise, and more basically of lifestyle choices should be emphasized for not only treatment strategies but preventive strategies. In fact, when lifestyle-related factors are evaluated with the healthy lifestyle index (HLI) involving diet and physical activity, women in the highest quintile of the HLI score had a significantly lower risk of EC (hazard ratio 0.61, 95% CI 0.51–0.72), underscoring the potential importance of lifestyle in lowering the risk of EC in premenopausal women [171].

## 9. Conclusions

This review article focuses on how metabolic disorders, especially obesity, are involved in EC. Specific TMEs are formed via excess estrogen produced by increased aromatase activity or decreased levels of SHBG, as well as chronic low-grade inflammation in adipose tissues with elevated levels of adipokines and cytokines, leading to insulin resistance. IGF activation due to decreased IGFBP via insulin resistance also promotes TMEs.

Hyperinsulinemia, rather than hyperglycemia, plays an essential role in the development of EC via the PI3K-AKT pathway. Over 90% of ECs have genomic alterations, causing enhanced insulin signaling to produce an optimal TME. We thus have a theoretical rationale for using PI3K inhibitors to treat EC. Clinical trials for recurrent or advanced ECs, however, have shown an unfavorable efficacy, and here we discussed the potential mechanisms. This failure is mainly due to activation of alternative pathways upon exposure to the inhibitors that can compensate for the PIK3-AKT pathway or to adaptive mechanisms including the insulin feedback pathway, thus limiting the efficacy of agents. Inhibiting these pathways will be a key to successful treatment, along with maintaining low levels of insulin during treatment. Combinations of PI3K-AKT inhibitors with specified diet protocols including the ketogenic diet are possible candidates for such strategies, and optimal combinations should be explored by ongoing and future clinical trials. Further understanding of specific TMEs via the insulin-PI3K pathway in obese women will provide openings for not only novel therapeutic strategies but preventive strategies against EC.

## Figures and Tables

**Table 1:** Representative clinical trials for endometrial cancer using metformin.

Author	Agents	Design	Number of EC Patients	Treatment	Representative Effects
Mitsuhashi et al. 2014 [134]	Metformin	Single arm	31	Preoperative	Tumor: Ki-67 ↓, phospho-rpS6↓, phospho-ERK1/2↓, phospho-AMPK↑. Serum levels: insulin↓, glucose↓, IGF-1↓. The ability of serums from patients to stimulate DNA synthesis decreased in cultured EC cells
Tabrizi et al. 2014 [135]	Metformin vs. MA	Non-blinded RCT	22 vs 21	Before histological assessment for uterine bleeding	Reversion to endometrial atrophy in 96% of patients treated with metformin, compared to 62% of patients treated with MA
Li et al. 2014 [136]	Metformin+EE+Cyproterone acetate	Single arm	5	Fertility-sparing, no operation	Reversion to normal endometrium in all patients
Laskov et al.2014 [137]	Metformin	Single arm	11	Preoperative	Tumor: Ki-67 ↓, phospho-rpS6↓. Serum levels: insulin↓, IGF-1↓, IGFBP-7↓
Schuler et al. 2015 [138]	Metformin	Single arm	20	Preoperative	Tumor: Ki-67 ↓, phospho-AKT↓, phospho-AMPK↓, phospho-rpS6↓, phospho-4E-BP-1↓, ER↓. PR →. Serum levels: free fatty acid↑.
Cai et al. 2015 [139]	Metformin vs. no treatment	Non-RCT	30 vs 30	Preoperative	Tumor: phospho-AMPK↑, phospho mTOR↓. Serum levels: IGF-1↓.
Sivalingam et al. 2016 [140]	Metformin vs. no treatment	Non-RCT	28 vs 12	Preoperative	Tumor: Ki-67 ↓, phospho-rpS6→, phospho-4E-BP-1↓, phospho AKT→, ER→, PR →, phospho ACC→, caspase 3→.
Soliman et al. 2016 [141]	Metformin	Single arm	20	Preoperative	Tumor: Ki-67 →, phospho-rpS6↓, phospho AKT↓, phospho-ERK1/2↓, phospho ACC→, caspase 3→. Serum: IGF-1↓, omentin↓, insulin↓, C-peptide↓, leptin↓.
Mitsuhashi et al. 2016 [142]	Metformin+MPA followed by Metformin alone	Single arm	29 (including 16 AEH)	Fertility-sparing, no operation	3-year recurrence-free survival in 89% of patients
Zao et al. 2018 [143]	Metformin vs. no treatment	Non-RCT	33 vs 32	Preoperative	Tumor: Ki-67 ↓, PI3K↓, phospho AKT ↓, phospho-S6K1↓, phospho-4E-BP-1↓.
Kitson et al. 2019 [144]	Metformin vs. placebo	Double- blinded RCT	45 (2 AEH, 43 EC) vs 43 (2 AEH, 41 EC)	Preoperative	Tumor: Ki-67 →, pIR →, IGF1R →. Serum: Glucose↑, Insulin→, HOMA-IR→, HbA1C→, IGF-1→, IGFBP-1→, Adiponectin→.
Petchsila et al. 2020 [145]	Metformin vs. placebo	Double- blinded RCT	25 vs 24	Preoperative	Tumor: Ki-67↓. No significant differences were detected in metabolic effects and adverse events between the metformin and the placebo groups.
Yang et al. 2020 [146]	Metformin+ MA vs. MA	Open-label RCT	76 (61 AHE, 15 EEC) vs 74 (62 AEH, 12 EEC)	Fertility-sparing, no operation	The CR rate within 16 weeks of treatment was higher in the metformin plus MA group than in the MA group (34.3 versus 20.7%, OR 2.0, 95% CI 0.89–4.51, *p* = 0.09) but the difference was more significant in AEH patients (39.6 versus 20.4%, OR 2.56, 95% CI 1.06–6.21, *p* = 0.04).

rpS6,ribosomal protein S6; ERK1/2, extracellular signal-regulated kinase-1/2; AMPK, AMP-activated kinase; IGF-1, insulin-like growth factor-1; EC, endometrial cancer; RCT, randomized controlled trial; MA, megestrol; EE, ethinyl estradiol;IGFBP-1/7, insulin-like growth factor binding protein-1/7; 4E-BP-1, eukaryotic initiation factor 4E-binding protein-1; ER, estrogen receptor; PR, progestreone receptor; mTOR, mammarian target of rapamycin; ACC, acetyl-CoA carboxilase; AEH, atypical endometrial hyperplasia; PI3K, phosphoinositide 3-kinase; S6K, S6 kinase 1; IR, insulin receptor; IGF1R, insulin-like growth factor receptor-1; HOMA, homeostasis model assessment-insulin resistance;OR, odds raio; CI, confidence interval.

**Table 2:** Representative clinical trials for EC using PI3K-AKT inhibitors in the main arm.

Author	Agents	Phase	Number of Patients	Cancer Type	Response	Molecular Markers	Hyperglycemia	Note
Slomovitz et al. 2010 [147]	Everolimus	II	35	Recurrent or advanced EC	SD 100%, CBR 22%	None	9%	
Oza et al. 2011 [148]	Temsirolimus	II	60	Recurrent or metastatic EC	chemotherapy-naïve group: PR 14%, SD 69%. chemotherapy-treated group: PR 4%, SD 48%	*PTEN* MT, pAKT, pmOR, pS6. (No correlation with response)	~5%	
Ray-Coquard et al. 2013 [149]	Everolimus	II	44	Advanced or metastatic EC	PR 5%, SD 32%	None	69%	Median PFS 2.8 months
Colombo et al.2013 [150]	Ridaforolimus	II	45	Recurrent or persistent EC	PR 11%, SD 18%	None	11%	6-month PFS 18%
Tsoref et al. 2014 [151]	Ridaforolimus	II	31	Recurrent or metastatic EC	RR 9%, SD 53%	PTEN, *PIC3CA* MT, *AKT1* MT (No correlation with response)	74%	
Fleming et al. 2014 [152]	Temsirolimus vs. Combination (Temsirolimus + Megesterol or Tamoxifen)	II	71	Recurrent or advanced EC	single: CR 6%, PR 16%, SD 52%, combination; CR 0%, PR 14.3%, SD 53%	pAKT, PTEN (No correlation with response)	18% (single), 14% (combination)	
Oza et al. 2015 [153]	Ridaforolimus vs. Comparator (Progestins or chemotherapy)	II (randomized)	64 vs. 66	Recurrent or metastatic EC	RR 0% vs. 4%, SD 35% vs. 17%	None	29% (Ridaforolimus), 3%(Comparator)	Median PFS 3.6 months (Ridaforolimus) and 1.9 months (comparator)
Emons et al. 2015 [154]	Temsirolimus	II	22	Recurrent or advanced EC	RR 10%, SD 25%	None	Not known	
del Campo et al. 2016 [155]	PF-04691502 vs. Gedatolisib	II (randomized)	18 vs. 40	Recurrent EC	Gedatolisib group; CR 3%, PR 13%, SD 24%, CBR 40%	Stathmin (low expression was correlated with higher CBR)	33% (PF-04691502), 0% (Gedatolisib)	PF-04691502 arm was discontinued early due to unacceptable toxicity
Banerji et al. 2108 [156]	AZD5363	I	59 (as expansion arm)	31 breast and 28 gynecologic cancers with *PIK3CA mutation* (including 10 ECs)	RR 4% in breast cancer, PR 8% in gynecologic cancer	All patients had *PIK3CA* MT	20%	
Myers et al. 2020 [157]	MK-2206	II	37	Recurrent EC	RR 11% (*PIK3CA* MT), RR 4%, (*PIK3CA* WT)	*PIK3CA* MT (No correlation with response)	31%	Median PFS 1.7 and 2.5 months with or without *PIK3CA* MT

EC, endometrial cancer; CR, complete response; PR, partial response; SD, stable disease; CBR, clinical benefit response; MT, mutation; PFS, progression-free survival.
